# Carboxymethylated Gums and Derivatization: Strategies and Significance in Drug Delivery and Tissue Engineering

**DOI:** 10.3390/ph16050776

**Published:** 2023-05-22

**Authors:** Madhuri Baghel, Kalyani Sakure, Tapan Kumar Giri, Sabyasachi Maiti, Kartik T. Nakhate, Shreesh Ojha, Charu Sharma, Yogeeta Agrawal, Sameer Goyal, Hemant Badwaik

**Affiliations:** 1 Department of Pharmaceutical Chemistry, Shri Shankaracharya Institute of Pharmaceutical Sciences and Research, Junwani, Bhilai 490020, Chhattisgarh, India; banchhormadhuri@gmail.com; 2 Department of Pharmaceutics, Rungta College of Pharmaceutical Sciences and Research, Kurud Road, Kohka, Bhilai 490024, Chhattisgarh, India; kalyanisakure88@gmail.com; 3 Department of Pharmaceutical Technology, Jadavpur University, Kolkata 700032, West Bengal, India; tapan_ju01@rediffmail.com; 4 Department of Pharmacy, Indira Gandhi National Tribal University, Amarkantak 484887, Madhya Pradesh, India; 5 Department of Pharmacology, Shri Vile Parle Kelavani Mandal’s Institute of Pharmacy, Dhule 424001, Maharashtra, India; kartik.nakhate@svkm.ac.in; 6 Department of Pharmacology and Therapeutics, College of Medicine and Health Sciences, United Arab Emirates University, Al Ain P.O. Box 15551, United Arab Emirates; shreeshojha@uaeu.ac.ae; 7 Department of Internal Medicine, College of Medicine and Health Sciences, United Arab Emirates University, Al Ain P.O. Box 15551, United Arab Emirates; charusharma@uaeu.ac.ae; 8 Department of Pharmaceutics, Shri Vile Parle Kelavani Mandal’s Institute of Pharmacy, Dhule 424001, Maharashtra, India; yogeeta.goyal@svkm.ac.in

**Keywords:** carboxymethylated gums, derivatization, drug delivery, tissue engineering

## Abstract

Natural polysaccharides have been widely exploited in drug delivery and tissue engineering research. They exhibit excellent biocompatibility and fewer adverse effects; however, it is challenging to assess their bioactivities to that of manufactured synthetics because of their intrinsic physicochemical characteristics. Studies showed that the carboxymethylation of polysaccharides considerably increases the aqueous solubility and bioactivities of inherent polysaccharides and offers structural diversity, but it also has some limitations that can be resolved by derivatization or the grafting of carboxymethylated gums. The swelling ratio, flocculation capacity, viscosity, partition coefficient, metal absorption properties, and thermosensitivity of natural polysaccharides have been improved as a result of these changes. In order to create better and functionally enhanced polysaccharides, researchers have modified the structures and properties of carboxymethylated gums. This review summarizes the various ways of modifying carboxymethylated gums, explores the impact that molecular modifications have on their physicochemical characteristics and bioactivities, and sheds light on various applications for the derivatives of carboxymethylated polysaccharides.

## 1. Introduction

Polysaccharides, which are requisite polymers found in plants, animals, fungi, and bacteria, are one of the leading trends in modern medicine [1,2]. Due to their nontoxicity, affordability, ease of availability, biosafety, biodegradability, and widespread regulatory approval, natural polysaccharides outperform synthetic ones in many ways [3,4,5]. Natural polysaccharides exhibit adequate biological properties but are less suitable as drug carriers than synthetic polymers, as their structure and property correlation revealed certain drawbacks. Some polysaccharides do not possess bioactivity due to their particular structure and physicochemical properties [6]. Bioactive polysaccharides have higher molecular weight, making cell membrane penetration challenging [7,8]. The bioactivities of some polysaccharides are quite low. Many of them have an uncontrolled hydration rate, viscosity loss, and thickening during storage, and are microbially sensitive [9]. They require some modifications because the structure of polysaccharides affects their bioactivities and physicochemical properties. The characteristic features and functional groups of polysaccharides facilitate the selective chemical or biochemical modifications of polymeric backbone that expands their applications [10,11,12].

Molecular modification is the process of altering the structure of a substance using physical, chemical, or biological methods to produce a variety of structural variants. With the right techniques, structural changes can be exploited to impact physicochemical and bioactive properties. The chemical modification of polysaccharides to boost their bioactivity has been highlighted in several recent studies [13,14,15,16].

It has already been revealed that there are various molecular modification strategies, such as sulfation [17], sialylation [18], phosphorylation [19], acetylation, alkylation, and others, including the most widely utilized carboxymethylation [20]. Modifying carboxyl, amino, or hydroxyl terminal groups with sulfate produced sulfated polysaccharides with improved biological activity [21]. Due to the limited variety of phosphate mono- and polysaccharides in nature, phosphate polysaccharides have few biological functions [22]. It has been discovered that monosaccharides such as fructose, glucose, and others lack intrinsic bioactivities that may be induced through phosphorylation modification. Under specific circumstances, selenite or selenious acid is frequently utilized to produce selenite–ester bonds with hydroxyl (-OH) groups on polysaccharides. By combining selenium with polysaccharide, one can create an organic complex that has the bioactivities of both substances while also being simple for the body to absorb and use [23]. Branches of polysaccharide molecules are primarily treated via acetylation modification, which results in a significant increase in polysaccharide solubility [24]. Acetic anhydride is converted into a positive electrophilic reagent capable of attacking polysaccharide molecules in this process. Decreased viscosity and improved solubility are two important factors in enhancing bioactivity, and these can both be achieved through alkylation modification at the main chain′s terminal end.

A polysaccharide chain is carboxymethylated (CM) by adding a carboxymethyl group to it. The Williamson ether synthesis serves as the foundation for the carboxymethylation reaction, in which carboxymethyl groups are etherified with primary or secondary alcohol groups of polysaccharides [25]. It is most typically used to improve the water solubility, viscosity, swellability, and bioactivity of polysaccharides. It provides structural diversity and a degree of crystallinity, enhances surface irregularity, imparts an anionic property, and even adds new bioactivities [26,27]. The low cost of chemical reagents, ease of processing, and nontoxicity of the products are the key advantages of this reaction [28]. For carboxymethylation, aqueous medium and organic solvent techniques are frequently employed [29]. Homogeneity in alkalization, minimal side reactions, a high etherification reagent usage rate, faster main reactions and etherification reactions, and process stability are all advantages of the solvent approach over the aqueous medium method. Isopropanol has the disadvantage of being both costly and harmful [30]. Carboxymethylation improves the polysaccharides’ solubility in water and decreases the viscosity, which enhances the free-radical initiator and monomer penetration during the grafting reaction, boosts mucoadhesiveness, and enhances reactivity.

Natural gums are polysaccharides comprising many joined sugar units that build large molecules [31,32]. Plants produce them as part of their injury recovery mechanisms. Gums are an industrial staple and superior to synthetic polymers because of their biosafety and biocompatibility [33]. Naturally occurring gums from plants, microorganisms, and seaweed present a remarkable potential for chemical derivatization and modification to create unique, cutting-edge biomaterials [34,35]. Among the carboxymethylated polysaccharides, carboxymethylated gums (CMGs) are most widely explored for their different functional characteristics and diverse medicinal applications. CMGs are used to formulate hydrogels [36], nanoparticles [37], tablets [38,39], nanohybrids [40], films [41], and microparticles [42] and also find applications in tissue engineering [43]. The structural and physicochemical characteristics of CMGs contribute to their capabilities in tissue engineering and drug delivery systems [44]. However, there are several issues with the carboxymethylation of natural gums that can be resolved by derivatization. These include the following factors: (1) Early gastrointestinal fluid erosion hinders the use of CMGs in sustained-release preparations for a longer time. Derivatization synergistically increases the sustaining capability [45]. (2) Derivatization increases thermostability as well as storage stability [46]. (3) An increase in the proportion of grafting contributes to a greater reduction in bacterial and fungal development [47]. (4) Derivatization increases the water affinity, swellability, and flocculation capability of the polymers [48,49]. (5) Additionally, CMGs have some limitations, including biodegradability, which severely limits their applications. With derivatization, these shortcomings can be overcome, giving the polymer framework novel properties (Table 1). Badwaik et al. explored the challenges and issues with the carboxymethylation of natural gum polysaccharides [44]. No review has been published to date that explores the derivatization of carboxymethylated gum derivatization technologies and their implications in drug delivery and other biological sectors. As a result, in the present review, we offer a systematic update on studies involving the chemical functionalization of carboxymethylated polysaccharides and their application in drug delivery, gene delivery, and tissue engineering.

## 2. Derivatives of Carboxymethylated Gums (CMGs)

CMGs have been modified to create biomaterials with superior characteristics. These alterations increase their partition coefficient, swelling capacity, thermosensitivity, flocculation ability, viscosity, and metal sorption properties, while others decrease viscosity and thermal stability. The derivatization of CMGs involves graft copolymerization, cross-linking, conjugation, and polyelectrolyte complexation.

### 2.1. Graft Copolymerization

The chemical modification of CMGs via graft copolymerization has prompted a great deal of interest in recent years and has made a significant contribution to the development of more effective applications in industrial and biological spheres. Graft copolymerization adds stearic bulk and protects the matrix and carbohydrate backbone [71]. Carboxymethylation improves gum grafting, as the carboxymethyl group of gums allows the monomer and initiator to diffuse. Additionally, the negative charge along the gum chain resulting from the ionizing tendency of carboxyl groups attracts initiators toward the gum, leading to the formation of additional active sites accessible to monomers and enhancing the gum’s reactivity. To initiate the grafting of monomers such as acrylonitrile, *N*-isopropylacrylamide, 4-vinyl pyridine, methoxy poly(ethylene glycol) amine (mPEG-A), methacrylic acid, acrylamide, 2-acrylamidoglycolic acid (2-AA-GA), etc., on carboxymethyl gums, initiator systems such as APS (ammonium persulphate), CAN (ceric ammonium nitrate), 1-ethyl-3-[3-(dimethylamino)propyl]-carbamide (E-DC) and N-HS (*N*-hydroxysuccimide), TMEDA (N,N,N,N′-tetramerthlene diamine) redox pair, bromated or thiourea redox pair, peoxymonosulfate (PMS) or thiourea redox pair, etc., have been produced. Graft copolymerization with microwaves has also been described.

#### 2.1.1. Graft Copolymerization Initiated by Free Radicals

**Figure 1:**
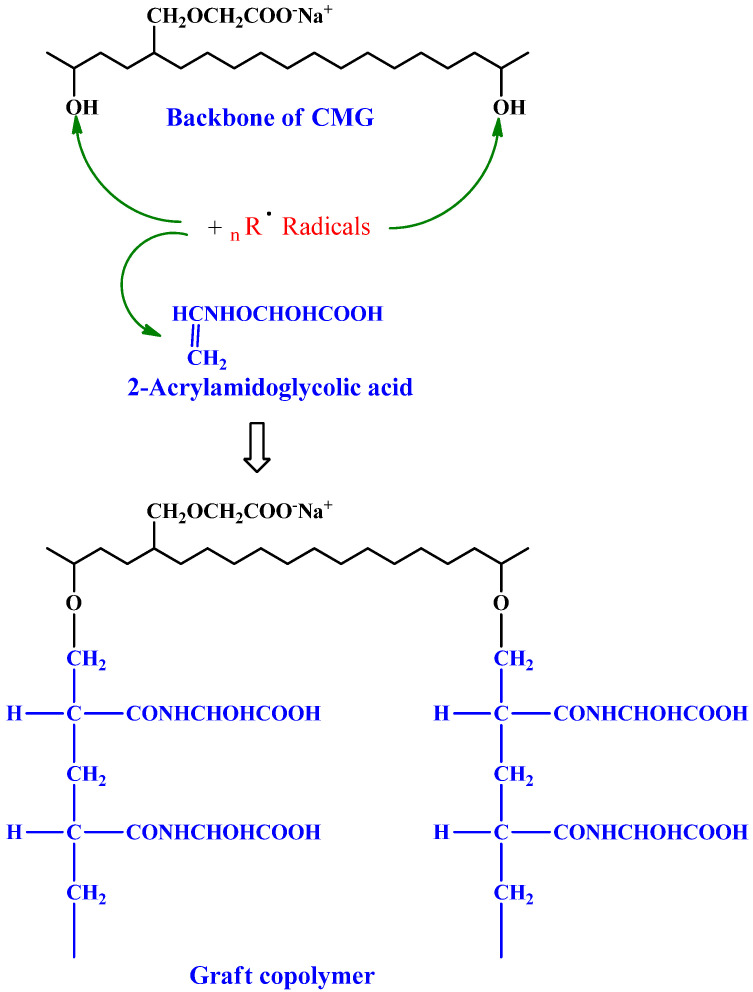
2-Acrylamidoglycholic acid graft partially copolymerized carboxymethylated guar gum. Reproduced with permission from [58]. Copyright© 2011, Elsevier Ltd.

Sand et al. grafted 2-AA-GA onto partially carboxymethylated guar gum (CM-GG) utilizing a redox pair, PMS, or thiourea (Figure 1) [58]. R1-SH (a protonated species) is generated when hydrogen ion interacts with thiourea, which further forms a complex via interaction with PMS. Dissociation produces R_1_-S and SO^4−^, which are responsible for abstracting hydrogen from CM-GG molecules. Monomer molecules acquire CM-GG radicals near the reaction site, enabling chain initiation, and they transfer free radicals to surrounding molecules, thus producing macroradicals. A graft copolymer is formed by connecting grafted chains. In comparison to CM-GG, grafted 2-AA-GA is more thermostable and has better metal ion sorption, swelling, and flocculation, properties. The long pendant chains of 2-AA-GA boost the graft’s swelling ratio. Access to coal particles is improved by dangling chains of 2-AA-GA.

Xia et al. produced KGM-g-mPEG (konjac glucomannan grafted methoxy polyethylene glycol) via the reaction of CM-KGM (carboxymethylated konjac glucomannan) with mPEG-A in the presence of E-DC and N-HS. For connecting mPEG to KGM, its hydroxyl group must be changed to an amino group [52]. From mPEG-OCOCH_2_NH-Boc, the Boc group was removed to make mPEG-OCOCH_2_NH_2_. E-DC permitted the nucleophilic attack of PEG’s primary amines on O-acylisourea to produce an amide bond. KGM-g-mPEG solubility increases with increased PEG content. The synthesized compound showed more than a 50% increment in the solubility of KGM (highest solubility; 17.80 g/100 mL in water). PEG-grafted KGM decreases solution viscosity at low shear rates. Li et al. produced KGM-g-mPEG, following the procedure described by Xia et al. [52], and formulated nanospheres utilizing α-cyclodextrin (α-CD).

Tripathy et al. grafted 4-vinyl pyridine onto CM-GG and examined its flocculation behavior, swelling, and metal ion sorption characteristics [50]. The generated isothiocarbamide radicals form thiourea and abstract hydrogen ions from the CM backbone of GGs to form free radicals. The graft copolymer chain is built by receiving CM-GG radicals, initiating the chain, and after propagation, finally leads to graft copolymer formation. The graft copolymerized CM-GG is thermally more stable (due to strong covalent connections between the grafted chains) and has better metal ion sorption ability (due to the poly-pendent chain). The coiling chain of poly(4-vinyl pyridine) absorbs more water due to the hydrophilic 4-vinyl pyridine monomer. H-partially CM-GG-g-methacrylamide (H-partially carboxymethylated guar-gum-grafted methacrylamide) was produced by Yadav et al. [49]. Deoxygenated potassium peroxy monosulfate was utilized to initiate the process. For alkaline hydrolysis, graft copolymers are reacted with varied concentrations of NaOH (1.5–3.5 N) [72]. Silver and potassium peroxy monosulfate are utilized to generate radicals [73]. N,*N*′-MB-AA (N,*N*′-methylene bisacrylamide) cross-links the grafted copolymer during the chemical process. Trivedi et al. performed acrylamide graft copolymerization utilizing CAN as a redox activator on the sodium salt of partially CM-GG [74]. Pal et al. produced polyacrylamide-grafted CM-GG using a free-radical activator, potassium persulfate [54]. The stimuli-responsive graft copolymer polysaccharides are formed by coupling (pN-IPAA) ((poly(N-isopropyl-acrylamide)) onto (CM-HP-GG) (O-carboxymethyl-O-hydroxypropyl-guar gum) in an aqueous solution, employing potassium persulfate and TMEDA as an initiating system [59].

For water-soluble polysaccharides or polymers, carbodiimide chemistry may be used in an aqueous solution, employing a coupling agent (E-DC) [75]. Thermosensitive graft copolymers with a CM-GG backbone and poly(*N*-isopropyl-acrylamide) (pNIP-AA) and pNIP-AA side chains were synthesized [49]. By combining CM-GG and pNIP-AA-NH_2_ with E-DC, CM-GG-g-pNIP-AA (carboxymethylated guar-gum-grafted poly(N-isopropyl-acrylamide) copolymers were made. By employing a chain transfer agent (2-amino-ethane-thiol hydrochloride), semi-telechelic pNPAA-NH_2_ homopolymers with a reactive group at one end were produced. CM-GG viscosity was elevated via hydrophobic modification using pNIP-AA. Researchers observed that E-DC alone has low coupling yields; therefore, adding N-HS improves efficiency [55]. The coupling reaction with the carboxyl groups of polysaccharides (carboxymethyl tamarind gum and CM-GG) and PEPO terminal amine in cold water utilizing E-DC or N-HS as coupling reagents was reported by Gupta et al. [60].

Soliman et al. produced amphoteric CM-CH-g-pAA (carboxymethyl chitosan-graft-polyacrylamide) copolymer using the peroxy graft copolymerization of acrylamide (AA) onto carboxymethyl chitosan (CM-CH) using potassium persulfate as initiator [56]. By employing potassium persulfate as an initiator, poly(*N*-vinyl imidazole) (pN-VI) grafting was carried out onto CM-CH in an aqueous solution [47]. Increasing potassium persulfate concentration reduces grafting yields. This could be due to initiator–chain-transfer agent competition or initiator–radical coupling. %G and %GE reach a maximum at a concentration of 1 mol/L, after which they further decline, suggesting that higher *N*-vinyl-imidazole (N-VI) concentrations do not stimulate additional grafting. Bamford and Schofield hypothesized a radical chain-transfer mechanism and degradative chain transfer to N-VI [76]. Furthermore, grafting a huge portion of polymer onto CM-CH generates a steric barrier for subsequent grafting. Sabaa et al. produced a novel superabsorbent polymer by graft copolymerizing 4-vinyl pyridine onto CM-CH chains in an aqueous solution using potassium persulfate as an initiator. Due to PVP’s basic pyridine group, grafted copolymers swell in acidic pH conditions. CM-CH-g-p4-VP (carboxymethylated chitosan-grafted poly(4-vinylpyridine)) has both basic (pyridine rings) and acidic (COOH) functionality; hence, at neutral pH, swelling is negligible. The presence of the -COOH group in CMCH’s major chains allows it to absorb Maxilon blue with better efficacy than Congo red.

#### 2.1.2. Radiation-Initiated Graft Copolymerization

Microwave (a radiation source) and potassium persulfate (an initiator) were used to produce polyacrylamide-grafted CM-GG [54]. Small-polar molecules such as water generate heat when microwaved due to the rotation of molecules. No free radicals are created. Whole-molecule rotation is prevented by larger molecules or macromolecules. When this happens, polar groups (such as the -OH groups attached to CMG molecules) absorb the microwave and are anchored to an immobile raft. Bonds collapse, creating sites for free radicals. Additionally, the microwave energy that is swiftly transmitted from water molecules to acrylamide molecules causes dielectric heating, which breaks double bonds and creates additional free radicals. The monomer and free radicals (produced on the polar -OH groups of the CMG backbone) recombine to create graft copolymers through chain initiation, propagation, and termination steps. Ammonium persulfate was used as a free radical activator in the microwave-assisted synthesis of carboxymethyl xanthan-gum-grafted polyacrylamide (CM-XG-g-pAA) copolymer. Synthesized nontoxic graft copolymers have better antibacterial efficacy than CM-XG [57].

Patra et al. grafted sodium carboxymethylated okra gum (Na-CM-OG) with polymethacrylamide (pMAA) using free-radical initiation and microwave radiation. The study used a redox free-radical initiator, CAN. When CAN is split up into Ce^4+^ ions, oxygen free radicals are created on the OG backbone, striking the -OH groups of the anomeric -CHOH groups of okra gum and removing the hydrogen atom via a redox process. pMAA radical is generated by combining pMAA with MAA free radicals. The study shows that carboxymethylation and graft copolymerization synergize okra gum’s sustaining capacity compared with either alone.

#### 2.1.3. Photo-Induced Graft Copolymerization

The graft copolymerization of the sodium salt of partially carboxymethylated guar gum (Na-PCM-GG) and acrylonitrile was carried out, utilizing ceric ammonium nitrate as the photoinitiator in an aqueous medium to create Na-PCM-GG-pMMA: a new graft copolymer [61]. The effects of various variables of synthesis were investigated, including the photoinitiator (CAN), monomer (AN), and nitric acid concentrations; in addition, the effects of the substrate amount, temperature, and reaction time on grafting yields were also investigated to determine the reaction conditions for the best possible photo-induced grafting. During alkaline hydrolysis, the nitrile groups of the appropriately synthesized graft copolymer Na-PCM-GG-g-pMMA could be rapidly converted into water-soluble carboxamide and carboxylate groups, accompanied by the in situ cross-linking of the grafted pMMA chains, yielding the superabsorbent hydrogel H-Na-PCM-GG-g-pMMA.

### 2.2. Cross-Linking

Cross-linking reduces polymer segment mobility and creates a three-dimensional network [77]. Carboxymethylation improves gums’ hydrophilicity and swelling in dissolution liquid and regulates drug release. Cross-linking reduces the gum’s swelling since the drug could leak out before reaching the absorption site [31]. Cross-linked polymers are sturdier than natural polymers. Cross-linking occurs when cross-linkers react with functional groups (-OH, -COOH, and -NH_2_) [66].

#### 2.2.1. Covalent Cross-Linking

**Figure 2:**
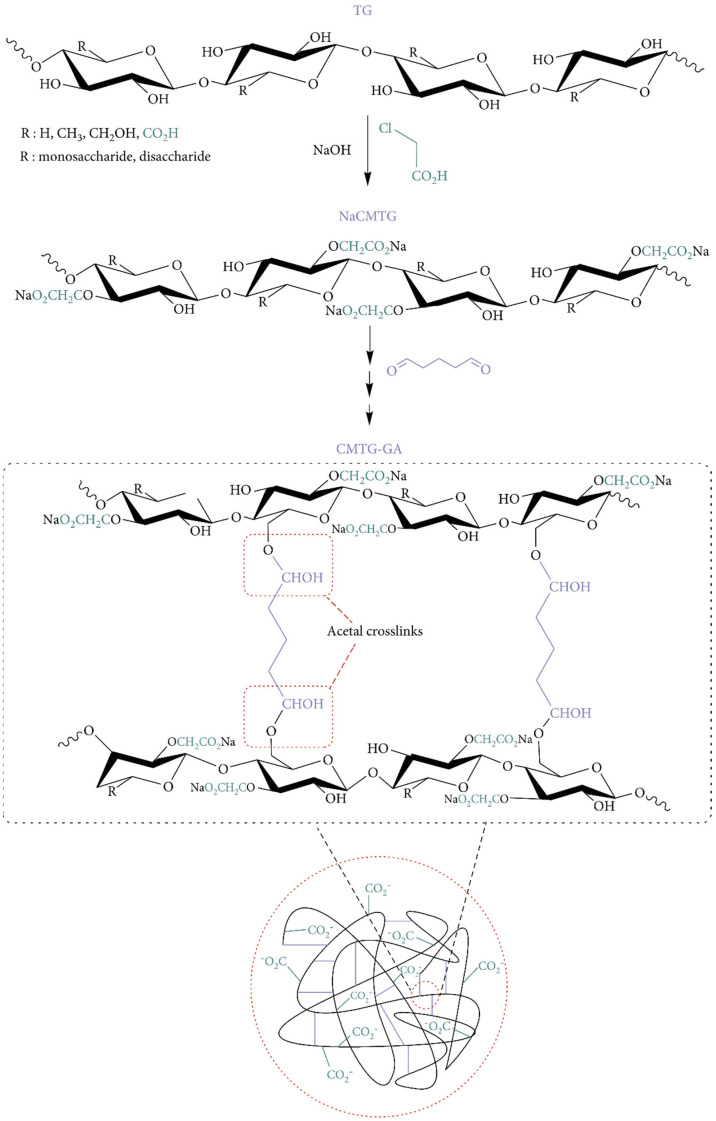
Proposed mechanism for synthesis of CM-TG-GA. Reproduced from [79]. Copyright© 2021, Yahya Bachra et al.

Silva et al. created carboxymethylated cashew gum (CM-CSG) and cross-linked cashew gum using epichlorohydrin [78]. NaOH forms polysaccharide alkoxide in the epichlorohydrin–polysaccharide reaction. Epichlorohydrin opens the epoxy ring, forming a new epoxy macromolecular. Cross-linking forms a three-dimensional network and reduces polymer segment mobility [56]. Carboxymethylation and cross-linking improved swelling by 23%. Cross-linking carboxymethylated tragacanth gum (CM-TG) polysaccharide backbones with glutaraldehyde (GA) produced CM-TG-GA hydrogels [79]. Figure 2 shows a probable process of CM-TG-GA synthesis. GA interacted with CM-TG hydroxyls to create hemiacetal cross-links. Finally, a three-dimensional superabsorbent polymer was developed. Cross-linking often results in unstable hemiacetal and acetal linkages between CM-TG and GA. This can form a semi-IPN (interpenetrating polymer networks) hydrogel. This instability aids in the biodegradation of superabsorbents after use.

The pH-sensitive delivery vehicle genipin-cross-linked O-CM-CH-GAR (O-carboxymethyl chitosan–gum Arabic) was explored by Guo-Qing Huang et al. [80]. Genipin, an aglycone formed from geniposide hydrolysis, reacts with the primary amine groups in CH. Due to its natural genesis, low toxicity, excellent biocompatibility, and cross-linking ability, genipin has been widely used in the cross-linking of targeted delivery systems [81]. O-CM-CH-GAR coacervates were cross-linked with genipin at pH 3.0, 4.5, and 6.0. Genipin cross-linking increased coacervate stability against pH sensitivity in simulated gastric fluid. In the simulated gastric solution, coacervates swelled more than in simulated colon and intestinal solutions (swelling was high at pH 4.5 and 6.0 than at pH 3.0). After 2 h of cross-linking, the cumulative percentages of the release of bovine serum albumin in a simulated gastric solution at pH 3.0, 4.5, and 6.0 microcapsules were 79.79%, 55.23%, and 17.14%, respectively.

The same authors created GA-cross-linked O-carboxymethyl chitosan–gum Arabic (O-CM-CH-GAR) coacervates and examined the effect of coacervation acidity on GA cross-linking behavior and cross-linking products. Cross-linking and GA sensitivity decreased from pH 3.0 to 6.0. GA cross-linking improved the flexibility and stability of O-CM-CH-GAR coacervates and acidity, thus affecting the swelling in simulated gastric fluid [82]. Coacervation acidity affected GA cross-linking in O-CM-CH–GAR coacervates and could be exploited for the intestine-targeted administration of sensitive substances. Polycation-to-polyanion ratios increased with an increase in coacervation pH, due to electrostatic interactions [83]. Different coacervate compositions may explain the variation in GA cross-linking activity.

#### 2.2.2. Ionic Cross-Linking

Ionic cross-linking is safer than covalent cross-linking since it is a simple and mild process. Cross-linker and gum derivatives interact ionically. Multivalent counter ions (e.g., calcium, barium, and aluminum) or anionic substances (e.g., sodium trimetapol phosphate) are needed for CMG ionic cross-linking. Anionic molecules produce electrostatic interactions, while metallic ions contribute to the establishment of coordinate covalent bonds.

Mocanu et al. [65] cross-linked carboxymethyl pullulan gum with sodium trimetaphosphate (Na-TMP) or epichlorohydrin. The Na-TMP cross-linked sample had highly acidic phosphate groups and weakly acidic carboxymethyl groups, while epichlorohydrin had just weakly acidic carboxymethyl groups. Cross-linking with Na-TMP improved hydrophilicity more than epichlorohydrin. Maity et al. synthesized and examined the influence of Ca^2+^ on the erosion, swelling, and mechanism of drug release from Ca-CM-XG matrices [84]. Ca-CM-XG matrices with more cross-linking swelled less and eroded more than CM-XG matrices. This is owing to calcium coordination with CM-XG carboxyl groups that increase gel layer viscosity. The simultaneous pH-dependent swelling and erosion affected drug release. Until reaching the critical calcium ion concentration, increased Ca^2+^ concentration enhances the drug release from matrices. Borax was successfully cross-linked with CM-GG. The borax cross-linked CM-GG solution (1% *w*/*v*) became viscous as a result of the cross-linking. In every testing medium, borax cross-linked CM-GG had a lower swelling index than CM-GG. Borax cross-linked CM-GG and CM-GG were employed as binders in the formulation of ibuprofen and paracetamol. Borax is really a well-known effective cross-linker for polymers with hydroxyl groups [85].

#### 2.2.3. Dual Cross-Linking

Ciprofloxacin HCl was incorporated into carboxymethyl sago pulp (CM-SGP). CM-SGP discs were created via cross-linking and irradiation [86]. To assess their potential as drug delivery systems, drug-loaded CM-SGP discs were characterized for size and weight uniformity, entrapment efficiency, drug loading, DSC (differential scanning calorimetric) studies, FTIR (Fourier transform infrared) spectroscopy, TGA (thermogravimetric analysis), X-ray diffraction, and FE-SEM (field-emission scanning electron microscopy). The swelling dynamics, in vitro release, and antimicrobial efficacy were determined. After exposure to radiation, ciprofloxacin′s antibacterial activity persisted; thus, CM-SGP discs have enormous potential as a method of administering medications to the eyes.

**Figure 3:**
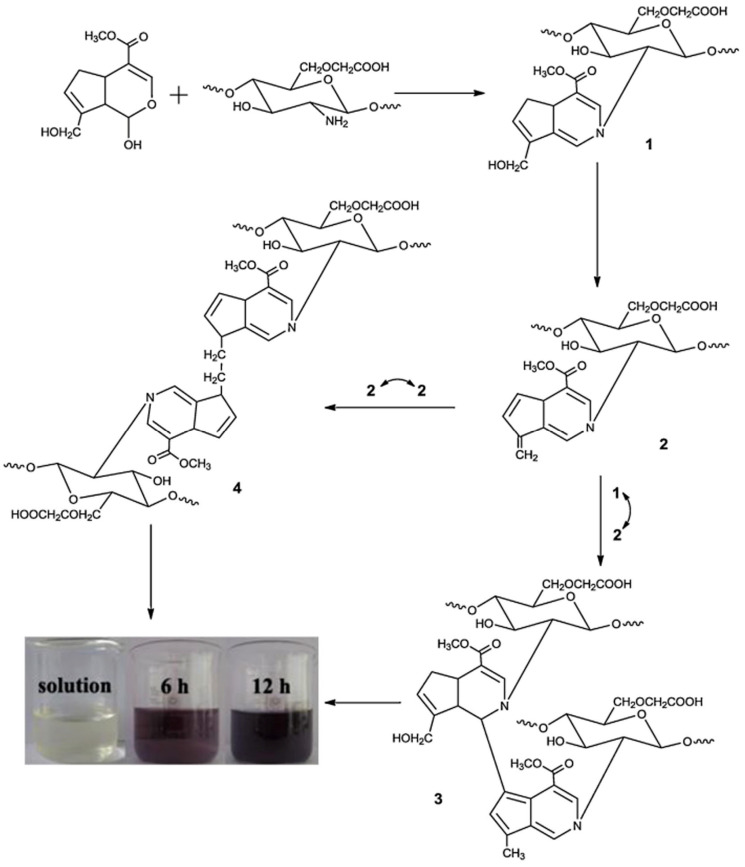
Mechanisms of CM-CGS–genipin cross-linking. Reproduced with permission from [87]. Copyright© 2014, Elsevier Ltd.

Carboxymethyl chitosan (CM-CH) and carboxymethyl cellulose (CM-C) were cross-linked with CaSO_4_ and genipin to make a hydrogel film via ionic and covalent cross-linking. Figure 3 depicts the mechanism of cross-linking. Being a nucleophilic reagent, genipin first reacted with amino groups of CMCS through a nucleophilic attack to produce the heterocyclic amino compound, the intermediate **1**. Following hydroxyl group elimination, another heterocyclic amino compound, the intermediate **2** was produced. These two intermediates subsequently covalently conjugated to dimers **3** and **4**, through the reaction of intermediate **1** with **2** and both intermediate **2**, respectively. After crosslinking with genipin, the CMCS amino groups were partly transformed as heterocyclic amines. Cross-linked CM-CH/CMC hydrogel films displayed swelling–deswelling characteristics with two major peaks at pH 3 and 7. The hydrogel films’ mechanical characteristics were also tested. Ionic and covalent cross-linking processes were found to affect the load and toughness of cross-linked hydrogels. The biocompatibility of cross-linked CM-CH/CMC films was shown by cells cultivated on cross-linked hydrogels and negative and positive controls [87].

Mitra et al. developed hydrogel beads via ionic and dual cross-linking by utilizing sequential and simultaneous techniques [88]. The mean dissolution time and drug diffusion coefficient demonstrate that the sequential method produces smaller beads with greater drug entrapment performance and prolonged drug release. Drug release decreased by increasing the cross-linking times and cross-linker concentrations. Drug release in the acid solution was accelerated by the solubility of the drug and the swelling of the matrices. Drug content, dissolution profiles, and FTIR demonstrated 3-month drug stability in the beads. Muniyandy et al. [89] combined CM-SGP with gelatin for extended drug delivery. GA-saturated toluene and aqueous aluminum chloride were cross-linkers for this study. GA has two aldehyde groups that cross-link hydroxyl polymers [82]. Coacervates encapsulated ibuprofen with 29–56% *w*/*w* loading and 85–93% *w*/*w* entrapment efficiency. Fresh drug-loaded coacervates were 10.8 ± 1.93 µm in size. Dual-cross-linked microcapsules released medication slowly over 6 h [82].

### 2.3. Conjugation

**Figure 4:**
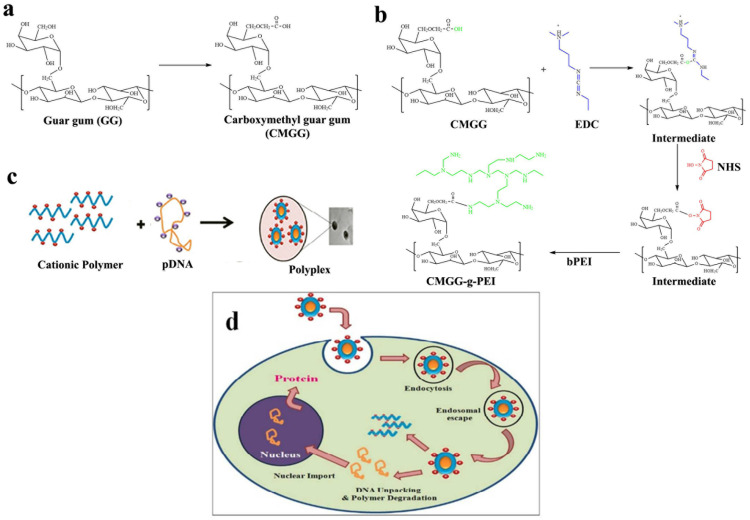
(**a**) Illustration of the production of CM-GG from guar gum; (**b**) CM-GG-g-PEI synthesis mechanism; (**c**) polymer–pDNA complex preparation; (**d**) schematic illustration of encapsulation of the polymer/DNA complex. Reproduced with permission from [90]. Copyright© 2011, Royal Society of Chemistry.

Ha et al. developed a novel cholesterol conjugate of carboxymethyl konjac glucomannan (CM-KGM) [63]. In aqueous media, these polymeric amphiphiles can aggregate by themselves. The CAC scores of the conjugates were lesser than 5.89 × 10^−3^ mg/mL, demonstrating the high aggregate-forming efficiency of the cholesterol residue. These self-aggregate display properties that depend on the pH and ionic strength. By using FTIR, 1HNMR, XRD, and zeta potential studies, the carboxymethylated guar-gum-grafted polyethyleneimine (CM-GG-g-PEI) copolymer was produced and described by Jana et al. [90]. When the plasmid DNA and polymer weight ratio was greater than 1:10, CM-GG-g-PEI showed strong binding to the plasmid DNA and generated complexes with diameters between 150 and 200 nm. The GG-grafted LMW-b-PEI (coupling low-molecular-weight branched PEI) copolymer was produced with CM-GG with LMW-b-PEI (weight 800 Da) using carbodiimide chemistry and N-HS as a coupling agent. Figure 4 depicts the CM-GG-g-PEI reaction scheme.

Researchers created a novel copolymer (carboxymethylated mesquite-gum-grafted polyethyleneimine (CM-MQG-g-PEI)) [91]. Initially, MQG carboxymethylation was carried out to form carboxymethylated mesquite gum (CM-MQG). The cross-linking of CM-MQG and b-PEI was then accomplished using the cross-linking agents EDC and N-HS. Carboxymethyl chitosan-conjugated magnetite (Fe_3_O_4_) nanoparticles (CM-CH-MG-NPs) were produced by covalently attaching CM-CH to the surface of MG-NPs through carbodiimide conversion in a medium of paraffin acetic acid [92]. CM-CH-MG-NPs had a spherical shape, with a diameter of 15 nm and a high CM-CH binding percentage (24.7 wt%). The capacity of magnetic sorbents to remove the heavy metal Pb(II) from aqueous systems was evaluated. Pb (II) sorption increased with pH (3.0–6.0) but reduced with C_NaNO3_ (0.001–0.500 M) or T (25–55 °C). CM-CH-MG-NPs sorb Pb(II) with a substantial Cs-effect. The Langmuir and Freundlich isotherms explain sorption equilibrium at each Cs (sorbent dosages). Langmuir-SCA and Freundlich-SCA isotherms described Cs effect data. The Cs effect was unaffected by pH, C_NaNO3_, and T.

### 2.4. Polyelectrolyte Complexation (PECs)

PECs are oppositely charged particle complexes (polymer/drug, polymer/polymer, and polymer/drug/polymer) and are formed when oppositely charged polyions interact electrostatically. Such contact may result in the precipitation, coacervation, or formation of the gel. This reduces the toxicity and other consequences of chemical cross-linking agents. These PECs meet biocompatible polymer system standards and can be tailored to produce active substance components and carrier substances. The Flory–Huggins theory concerning the free polyelectrolyte energy and electrostatic forces resulting from mixing has been postulated to explain PEC formation. Two polymers’ backbones typically repel one another, but how they interact depends on their charge fraction. The Flory interaction parameter, i.e., polymer backbone repulsion, prevails when the charge fraction is low, causing the solution to separate into two phases. At high charge fractions, polymers form a complex due to electrostatic interactions.

The carboxylic group gives the gum its anionic properties and enables the formation of PECs with cationic polymers. Chitosan has an overall positive charge because of the amino groups it contains, making it simple to combine with polymers that have a negative charge. The reaction of CMG and CH PECs can be illustrated by:

$$CMG-{COO}^{-}+{CH-{NH}_{3}}^{+}\to {CMG-COO}^{-}{{NH}_{3}}^{+}-CH$$

In a previous study, chitosan (CH) and carboxymethyl gum katira (CM-GK) interaction resulted in the development of PECs that were modified using ofloxacin as the reference drug [93]. The results of that study demonstrated that increasing the ratio of CM-GK and CH in the CM-GK-CH-PECs (carboxymethyl gum katira–chitosan PECs) decreased the percentage yield while increasing the percentage of drug entrapment. Drug loading at 50% (%*w*/*w*) and polymer ratio (CM-GK/CH) 2.13 were the best calculation parameters. The optimized batch of the CM-GK-CH-PECs had an ofloxacin entrapment efficiency of 84.86% and a yield of 69.04%. Furthermore, using Higuchi’s square-root kinetics, the improved batch of CM-GK-CH-PECs released ofloxacin at 84.32%. The conclusion drawn from this work is that the polyelectrolyte complex of CM-GK and CH offers significant promise as a polymer-based method for sustained drug administration.

Chitosan and carboxymethyl cashew gum compounds were developed, and their thermal stability was assessed by Maciel et al. [94]. Carboxymethyl konjac glucomannan [95], carboxymethyl tamarind kernel powder [96], carboxymethyl fenugreek gum [97], carboxymethyl guar gum [98], carboxymethyl gum kondagogu [99], and cashew gum have been complexed with chitosan to form PECs and utilized in various drug delivery system.

### 2.5. Esterification

**Figure 5:**
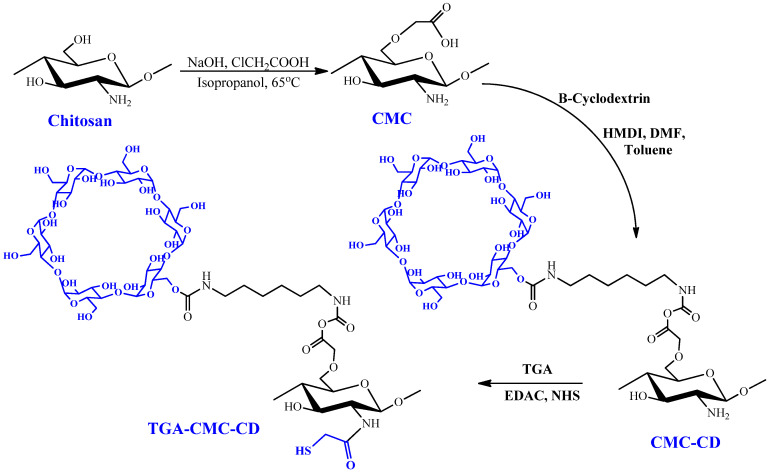
Chitosan modification and production of the TGA-CM-CH-CD polymer. Reproduced with permission from [100]. Copyright© 2013, Springer Science Business Media, New York.

Using thioglycolic acid, More et al. esterified carboxymethyl gellan gum (CM-GLG). The hydroxyl group of the glucuronic acid portion of CM-GLG is esterified with the carboxyl group of thioglycolic acid during the esterification reaction [64]. As shown in the scheme in Figure 5, thiolated carboxymethyl chitosan-β-cyclodextrin was produced by covalently attaching thioglycolic acid to the primary amino groups of carboxymethyl chitosan-β-cyclodextrin (CM-CH-β-CD) [100]. Another study used a two-step process to synthesize thiolated CM-CH-β-CD. In the first stage, EDC and N-HS were used to form an amide link between the main amino groups of CM-CH and β-CD. The carboxyl groups of the resulting CM-CH-β-CD were coupled with the amino groups of cysteine methyl ester-hydrochloride in the second step [70].

## 3. Applications of Derivatized Carboxymethylated Gums

### 3.1. Carrier for Drug Delivery

Drug delivery systems that involve derivatized CMGs include oral, ocular, and transdermal delivery (Table 2). Derivatized CMGs have been studied in a variety of therapeutic oral delivery systems that are commonly used for drug administration, either as controlled or extended-release matrices or as useful biomaterials. Derivatized CMGs have been used to transport medications to a particular site, including the colon, small intestine, stomach, and mouth.

#### 3.1.1. Oral Drug Delivery

The genipin-cross-linked O-CMC-GA coacervate was investigated for a pH-sensitive delivery system. Both the acidity of coacervate and cross-linking duration impacted O-CMC–GA pH responsiveness and BSA releases [80]. Due to their stability and gradual expansion in simulated gastrointestinal fluids; GA-cross-linked O-CMC-GAR coacervates potentially carry sensitive substances to the intestine. Coacervation acidity affected GA cross-linking behavior, and the product attributes are also changed by modifying the coacervate composition [82].

Patra et al. produced a prolonged-release tablet matrix for sodium carboxymethyl okra-gum-grafted polymethacrylamide (Na-CM-OG-g-PMA) copolymer [45]. The diclofenac sodium tablet formulation with S-CMOG-g-PMA with DCS-0.604 and %G-423.4 exhibited remarkable prolonged drug release (90% for 11.7 h) and a 72.0 similarity factor. The copolymer proved advantageous in terms of biodegradability and biocompatibility, rendering it a sensible semi-synthetic biopolymer. Carboxymethyl fenugreek galactomannan–gellan gum–calcium silicate (CMC-Fg-Gm-GG-CS) hydrogel beads were made to transport glimepiride in a controlled manner. Ionotropic gelation with Al^+3^/Zn^+2^/Ca^+2^ ions produced glimepiride-loaded hybrids. Formulations showed sustained drug release (62–94% for Q8hrs) and good drug encapsulation efficiency (EE; 48–97%) [97].

In order to generate CM-XG-g-pAA, Badwaik et al. synthesized carboxymethyl xanthan gum and then further grafted it with polyacrylamide. Wet-granulated tablets release the drug gradually. The release profile followed Korsmeyer–Peppas equation and showed drug transport via erosion and diffusion [105]. Bajpai and Sharma formulated vitamin-B12-loaded barium ions in sodium alginate CMC-GG hydrogel beads. Encapsulation efficiency was 50%, and the release of drug in the simulated gastric fluid was 20% (in 3 h), and that in the simulated intestinal fluid was 70% (in 7 h) with vitamin-B12-loaded beads [106].

Colon-specific delivery systems are important for treating ulcerative colitis and irritable bowel disease, as well as for the systemic injection of protein and peptide drugs [98,109]. Maity and Sa investigated cross-linked carboxymethyl xanthan gum tablets for prednisolone delivery. Ca^2+^ ion modulation regulates carboxymethyl xanthan gum drug release from matrix tablets [84]. Cross-linking limits polymer chain flexibility by generating a gel layer around the tablet’s surface. Increased cross-linking agent density enhances gel strength and reduces macromolecular mesh size, reducing drug diffusion through the gel layer. When placed in the colon, the optimized formulation released the drug in 10 h [109].

In another work, Kumar et al. produced a fluticasone colon-specific tablet matrix using cross-linked chitosan and CM-GG/IPC (interpolymer complexes). Chitosan:CMG (50:50) tablets encapsulated with IPC inhibited drug release in the small intestine and stomach, and it also delivered the drug to the colon [98]. Singh et al. produced metronidazole matrix tablets by cross-linking CM-GG with Ca^2+^ ions. Ca^2+^ ion concentration improved gel layer viscosity and inhibited water entry, minimizing tablet swelling and drug release. After a certain concentration of Ca^2+^ ions, the gel layer’s viscosity decreased as a result of matrix erosion. After a certain Ca^2+^ concentration, matrix erosion reduced the gel layer’s viscosity [104]. Randhawa et al. produced colon-specific targeted tamoxifen pills by covering them with chitosan and carboxymethyl fenugreek gum (CM-FG) or chitosan and CM-GG. The medication was released from the coated tablets at pH 6.8 while being protected from absorption in the stomach and small intestine [39].

A significant amount of effort has been put towards enhancing the mucoadhesive properties of polymeric carriers to boost drug absorption [110]. Because mucoadhesive delivery systems remain at the site of drug absorption for a longer period of time, dosage frequency can be reduced, thus increasing patient compliance. Mucoadhesive polymers rely on hydrogen bonding and ionic interactions with mucus [106]. Thiol-containing polymers have greater adhesion. Thiomers engage cysteine-rich mucus glycoproteins via disulfide exchange. Poor interactions between thiolated polymers and hydrophobic drugs lead to rapid drug release, limiting their clinical usefulness. Grafting produces thiolated carboxymethylated gums with modulated drug release [100].

Prabaharan et al. synthesized thiolated carboxymethyl chitosan-g-β-cyclodextrin (Th-CM-CH-g-β-CD) for ketoprofen [70]. Similarly, thiolated carboxymethyl chitosan-g-β-cyclodextrin nanoparticles (Th-CM-CH-g-β-CD) with maximum mucoadhesion and albendazole entrapment efficiency were prepared using an aqueous solution containing tripolyphosphate (1 wt%) and the polymer (115.65 lmol/g) of grafted thiol groups for the controlled release of hydrophobic drugs [95]. Anionic gelation with sodium tripolyphosphate produced TGA-CMC-CD nanoparticles for mucosal albendazole delivery. More et al. studied the mucoadhesive capability of carboxymethyl gellan gum–thioglycolic acid (CM-GG-TGA) conjugate and its physicochemical properties [69].

#### 3.1.2. Ocular Delivery

Ciprofloxacin HCl is the preferred medication for treating corneal ulcers and bacterial conjunctivitis, but its usability is limited by a relatively shorter elimination half-life [96]. Ordinary ophthalmic ointments and drops require multiple administrations to maintain plasma concentration. Therefore, ciprofloxacin HCl in a controlled-release dosage form may increase ocular bioavailability, lessen the administration frequency, and prolong the amount of time the drug remains in the precorneal tissue. Carboxymethyl sago pulp discs show potential in the ophthalmic delivery of ciprofloxacin because they can enhance ocular bioavailability by prolonging the time the drug is interacting with corneal tissues and by removing the need for recurring administration, as is necessary for conventional eye drops. The administered dose also satisfies the sterilizing condition, a necessity for ocular drug administration [86].

#### 3.1.3. Transdermal Delivery

**Figure 6:**
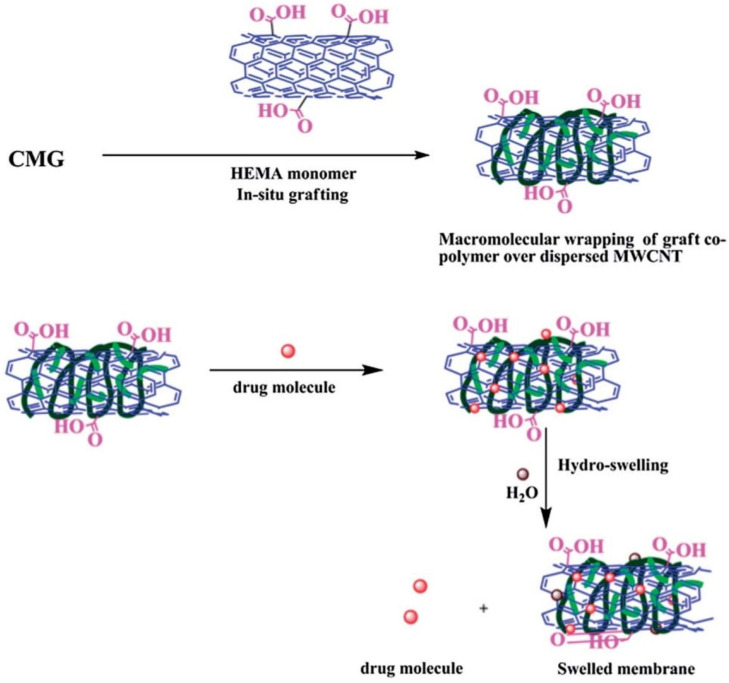
Schematic presentation of HEMA-grafted CMG (wrapping over the anisotropic MW-CNT units to produce an effective encapsulate for the drug and ensure its steady release over a prolonged time). Reproduced with permission from [111]. Copyright^©^ 2014, Royal Society of Chemistry.

Multiwalled carbon nanotube (MW-CNT) in situ composite membranes complexed with 2-hydroxyethyl methacrylate (HEMA)-grafted CM-GG were used to develop a transdermal device for the controlled release of diclofenac sodium. Supreme matrix interactions with 0.5 and 1 wt% MW-CNT resulted in a superior copolymer-adsorbed fibrillar alignment of MW-CNT compared with 2 and 3 wt% MW-CNT. These formulations improved encapsulation efficiency and prolonged drug release (65% vs. 17% at 1 wt%). It was determined that each strategy significantly lengthened the drug molecules’ half-lives (from 2.5 h at 1 wt% to 47 h). The microstructure wrapped in a copolymer is graphically depicted in Figure 6 [111]. Similarly, Giri et al. produced hydrogels consisting of chemically modified multiwalled carbon nanotubes (MW-CNTs) and carboxymethyl guar gum (CM-GG) for prolonged and consistent release of diclofenac sodium transdermally [107].

#### 3.1.4. Vaccine Delivery

**Figure 7:**
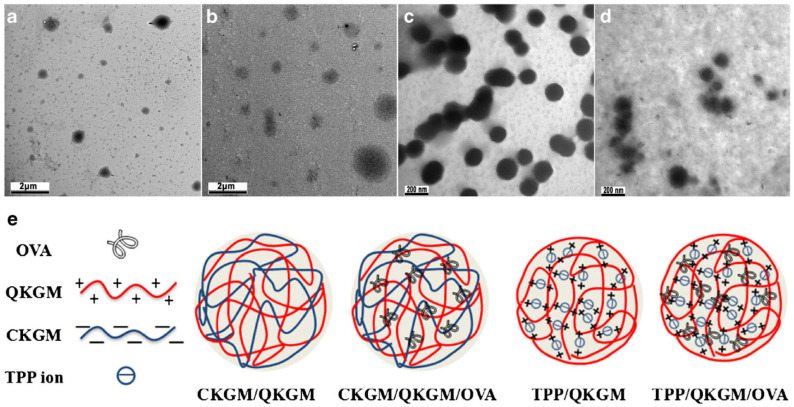
TEM images of blank and modified konjac glucomannan NPs and respective OVA-loaded NPs: (**a**) blank CM-KGM/Q-KGM/OVA NPs; (**b**) blank TPP/Q-KGM NPs; (**c**) TPP/Q-KGM/OVA NPs; (**d**) image of various kinds of NPs; (**e**) the schematic illustration of various kinds of NPs. Reproduced with permission from [101]. Copyright© 2018, Springer Science Business Media, LLC, part of Springer Nature.

In an interesting study, Chen et al. chemically modified konjac glucomannan (KGM) to generate anionic CM-KGM and cationic quaternized konjac glucomannan (Q-KGM). Two different types of nanoparticles (NPs), namely sodium tripolyphosphate/Q-KGM and CM-KGM/Q-KGM, were developed. To further investigate the impact of NPs on the immune response in mice, the prepared NPs were loaded with ovalbumin (OVA). Figure 7 shows a diagrammatic representation and TEM images of the formulated NPs. The NPs had an asymmetrical spherical morphology and excellent sustained-release characteristics. It was also discovered that the OVA-loaded NPs were not toxic to cells. Additionally, the nanoparticles produced from modified KGM could boost immune system activity and improve both the humoral and the cellular immune response [101]. Shi et al. also fabricated nanospheres from CM-KGM and 2-hydroxypropyl trimethylammonium chloride chitosan (HP-TmAC) loaded with OVA for vaccine delivery [102].

#### 3.1.5. Enzyme Encapsulation

Grafted CMGs are frequently used to create nanospheres as carriers for delivering drugs. To enclose the enzyme glucose oxidase in an aqueous solution, Li et al. produced self-assembled rod-coil carboxymethyl konjac-glucomannan-grafted poly(ethyleneglycol) (CM-KGM-g-PEG) and α-CD (α-cyclodextrin) complexes [46]. In comparison to free, unencapsulated glucose oxidase, the entrapped form had superior storage stability, optimum bioactivity over a broader pH range, and better thermostability. Additionally, it demonstrated in vitro biocompatibility. Similar research was conducted by Mocanu et al. on the interactions of biologically active compounds, including drugs (propranolol and quinidine), enzymes (lysozyme), and carboxymethyl pullulan microparticles cross-linked with siloxane [103]. The same group of authors produced the side chains of Jeffamine-[polyoxy-alkyleneamines (polyethylene-oxide)] units in cross-linked carboxymethyl pullulan hydrogels. These hydrogels have thermoassociated capabilities because of the Jeff units and pH-sensitive features because of the anionic functional groups. Interactions between proteins such as bovine serum albumin and lysozyme, as well as antioxidants such as lutein, and these hydrogels were induced to determine their suitability for use in drug delivery.

### 3.2. As a Gene/Vaccine Delivery Vehicle

Gene delivery vehicles can be either viral or nonviral vectors. Interest in nonviral gene delivery vehicles has increased due to the potential infectivity, sophisticated and complex production, immunogenicity, and carcinogenic effects of viral vectors. To increase effectiveness and lessen the cytotoxicity of gene vector transfection, the cross-linking of low-molecular-weight units with degradable connections has been studied [112]. A nonviral vector with high buffering capacity and transfection effectiveness is low-molecular-weight branched polyethyleneimine (LMW b-PEI) [113]. This polymer has high charge density, the potential to destabilize membranes, and the capacity to shield endocytosed plasmid DNA from enzymatic deterioration, rendering it ideal for encasing plasmid DNA in nanoparticles for gene therapy. PEI’s toxicity increases with molecule weight and thus is considered a risk for gene delivery [114,115]. These polymers do not break down in the body and pose a long-term threat [116]. To alleviate this limitation, a GG-grafted LMW b-PEI (800 Da) copolymer was produced and tested. CMGG-g-PEI buffered better than GG. The produced copolymer condensed pDNA by producing positively charged polyplexes, and it shielded pDNA from DNase I digestion and allowed it to enter the cell via its higher electron sponge effect (Figure 4). In in vitro experiments, the transfection of A549 cells using CMGG-g-PEI/plasmid DNA complexes was superior to that using LMW b-PEI (800 Da)/pDNA complexes and inferior to that using b-PEI (25 kDa)/plasmid DNA complexes. When compared to PEI, the graft copolymer showed much lower cytotoxicity. Hence, it has been established that the nonviral gene therapy vector CM-GG-g-PEI is a reliable and effective treatment option [90].

### 3.3. Therapeutic Applications

Sabaa et al. developed antibacterial CM-CH-g-PNVI copolymers [47]. Gram-negative (*E. coli*) and gram-positive bacteria (*S. aureus*) were exposed to CMCh-g-PNVI and chitosan. *S. aureus* viable cell count was inhibited more by chitosan-g-PNVI (G-107%) than by CM-CH-g-pNVI (G-99%), with 61.7% vs. 46.7% inhibition. Chitosan-g-PNVI inhibited *E. coli* by 74.1%, whereas CM-CH-g-pNVI inhibited it by 93.1%. Increasing the graft percentage led to an increase in the inhibition of *E. coli* and *S. aureus* viable cell numbers. Chitosan and its high-molecular-weight derivatives cover cell surfaces and inhibit intracellular leakage [117]. Likewise, chitosan-g-PNVI and CM-CH-g-pNVI analogues were tested on *A. fumigatus* and *F. oxysporum*. The 99% PNVI graft on CMCh inhibited *F. oxysporum* and *A. fumigatus* by 48.6% and 38.5%, respectively. Increasing the graft proportion improves inhibitory activity. CM-CH-g-pNVI (G-197%) inhibited *F. oxysporum* and *A. fumigatus* by 63.3% and 54.0%, respectively. Orsu et al. created nanofiber films from the biopolymer carboxymethyl tamarind gum (CM-TG), which was potentiated with reduced graphene oxide (rGO) and polyvinyl alcohol for the potential proliferation of neural cells. The results showed exceptional neuronal growth through an additive effect, which is important for replicating the extracellular matrix (ECM) and can be clinically tested for a variety of neurodegenerative illnesses [118].

In another investigation, the antibacterial activity of XG, CM-XG, CM-XG-g-PAAm, and m(CM-XG-g-PAAm) against *S. aureus* and *E. coli* was studied [84]. The carboxymethylation of XG improves water solubility. Antibacterial CM-XG may release H^+^ ions that penetrate the bacterial cell walls and induce cell death. Acrylamide grafting improves CMXG’s solubility and cationic centers. Synthesized graft copolymer’s antibacterial activity may be owing to the interactions between CM-XG-g-PAAm’s positive charge and *E. coli* (lipopolysaccharide) negative charge. Grafted carboxymethylated mesquite gum demonstrated improved hemocompatibility over b-PEI and carboxymethylated mesquite gum (CBX-MG). A hemolysis test was designed to assess polymer erythrocyte lysis [111]. The outcomes demonstrated nonhemolytic properties at doses less than 0.1 µg/mL (CBX-MG-PEI). The copolymer’s physicochemical and hemocompatibility potentials render it useful in formulating nanoparticles, transfection, and biomaterials [91].

### 3.4. Tissue Engineering

Tissue engineering can repair organs and tissues. Patient cells are seeded and cultivated in vitro to generate a new organ or tissue. An injectable, biodegradable hydrogel system including the conjugates of chondroitin sulfate tyramine (CDS-TY) and carboxymethyl pullulan tyramine (CM-PL-TY) was produced under physiological circumstances with horseradish peroxidase (HR-PX) and hydrogen peroxide (H_2_O_2_) for cartilage tissue engineering (CT-TE). CT-TE is a viable therapeutic strategy for cartilage regeneration and has advantages over existing cartilage treatment methods [119]. For regenerative medicine, this injectable method is effective, intrusive, and readily adaptable. The physicochemical features of a stable hydrogel system can be modified by adjusting the polymer weight ratio and cross-linking reagent concentrations. Porcine auricular chondrocytes encapsulated in CMP-TA and CS-TA hydrogels showed high cytocompatibility. CMP-TA and CS-TA composite hydrogels showed improved cartilaginous ECM deposition and cell proliferation, which facilitates chondrogenesis. A mouse subcutaneous implantation model’s histological analysis confirmed the hydrogel system’s tissue compatibility. Injectable pullulan/chondroitin sulfate composite hydrogels may be effective for rebuilding cartilage tissue. Bao et al. created new hydrogel films from CMCS and CMC, which were double-cross-linked using genipin and CaSO_4_ as ionic and covalent cross-linkers, respectively [87]. The main effects on the cross-linked hydrogels’ toughness and maximum load were caused, respectively, by ionic and covalent cross-linking. The biocompatibility of the cross-linked hydrogel films manifested in cultured cells, as well as negative and positive controls. Due to their advantageous characteristics, the double-cross-linked hydrogel films could be used as a biomaterial for skin tissue engineering.

## 4. Conclusions

Current developments and research tactics are focused on functionalizing established materials. One of the most studied conversions that lead to the discovery of newer biomaterials with extremely potential applications is modifying carboxymethylated gums. However, the application of carboxymethylated gum has encountered some challenges. Early gastrointestinal fluid erosion hinders the use of CMG in sustained-release preparations for a longer time. Derivatization, however, synergistically increases the sustaining capability. Thermostability and storage stability are also increased by derivatization. An increase in the proportion of grafting contributes to a greater reduction in bacterial and fungal development. Derivatization increases the water affinity, swellability, and flocculation capability of polymers. Additionally, CMG has some limitations including biodegradability, which severely limits its applications. With derivatization, these shortcomings can be overcome, providing the polymer framework with novel properties.

The prospective implications of modified carboxymethylated gums with respect to physiochemical characteristics and their biomedical and pharmaceutical applications are discussed in this review, with an emphasis on drug delivery, in vitro cell culture, and tissue engineering. For these uses, the biocompatibility of modified carboxymethylated gums and their simplicity in producing derivatives with novel characteristics are their most intriguing qualities. Almost all types of bioactive compounds can be prepared using modified carboxymethylated gums for prolonged and controlled release. Altogether, the data on the uses of derivatized carboxymethylated gums are promising. More research is needed before derivatized carboxymethyl-gum-based carriers may be used in clinics.

## Figures and Tables

**Table 1:** An overview of how derivatization affects CMG characteristics.

SN.	Modification	Effect	References
1.	Graft copolymerization with 4-vinyl-pyridine	Greater degrees of swelling capacity An improved coefficient of partitioning Improvements in the characteristics of metal uptake Improved flocculation capacity Decreased thickness or viscosity Enhanced antimicrobial activity	[48,50,51]
2.	Graft copolymerization with poly-(ethylene glycol)	Improved aqueous solubility Decreased thickness or viscosity Improvements in the efficacy of enzyme encapsulation	[46,52]
3.	Graft copolymerization with poly-(*N*-isopropyl acrylamide)	An increase in viscosity Thermosensitive characteristic Thermothickening behavior is absent	[53]
4.	Graft copolymerization with poly-acrylamide	Enhanced flocculation potential High color removal efficacy Greater thermostability Improved flocculation characteristics	[54] [55] [56] [57]
5.	Graft copolymerization with 2-acryl-amidoglycolic acid	Increased swelling threshold Increased metal sorption capacity Improved flocculent Better resilient in the heat Resistance to biodegradation	[58]
6.	Poly-(*N*-vinyl imidazole) grafted with (PEPO) [Poly-(ethylene oxide-co-propylene oxide)]	Enhanced thermostability Enhanced antibacterial effectiveness More elastic-like behavior Increased viscosity	[59] [60]
7.	Graft copolymerization with polymethacrylamide	Increased water uptake Enhanced sustained-release capacity	[45]
8.	Graft copolymerization with acrylonitrile	Improved swelling property	[61]
9.	Graft copolymerization with methacrylate	Increased water-holding capacity Soil conditioner	[62]
10.	Alterations involving cholesterol	Behavior of self-aggregation Dependency on pH and ionic strength	[63]
11.	Jeffamine substitution	Sensitive to temperature Sensitive to change in pH	[36]
12.	Barium ions cross-linked bipolymeric blend with sodium alginate	Swelling that depends on pH	[64]
13.	Cross-linking with calcium ions	Swelling, erosion, and drug release depend on pH	[65]
14.	Cross-linking with Epichlorohydrin	Better ability to swell Better temperature stability	[66]
15.	Cross-linking with polyelectrolyte complexation with chitosan	Less thermal stability	[67]
16.	Cross-linking with citric acid	Increased tensile strength of hydrogel Wound-healing activity	[68]
17.	Thiolation	Increased strength of mucoadhesion	[69]
18.	β-cyclodextrin grafting followed by thiolation	Improved mucoadhesive properties Controlled rate of release Improved water absorption	[70]

**Table 2:** Summary of derivatized CMGs utilized as drug delivery carriers.

S. No.	Drug Delivery	Derivatized Gum	Drug	Comments	Reference
1.	Intestine-targeted delivery system (tablet)	Genipin-cross-linked O-CMC–gum Arabic coacervates	BSA	pH-sensitive delivery BSA release in the gastric fluid solution at 4.5 and 3.0 was substantially more than those created at pH 6.0. In three different simulated solutions, the average BSA release percentages were 79.79%, 55.23%, and 17.14%. It bypassed the stomach’s acidic environment and released drugs in the intestine.	[80]
2.	Intestine-targeted delivery system (tablet)	GA-cross-linked O-CMC–gum Arabic coacervates	--	It provides a matrix that is both consistent and resistant to erosion. Sustainable and controlled release pH-sensitive delivery The swelling ratio of coacervates was greater at a pH of 1.2 than 6.8.	[82]
3.	Sustained-release drug delivery (monolithic matrix tablet)	Sodium carboxymethyl okra gum-grafted polymethacrylamide copolymer	Diclofenac sodium	Smart semi-synthetic biopolymer Sustained release carrier (90 % drug-release at 11.7 h) The grafting percentage was 644.1 and the extent of carboxymethyl modification was 0.604 ± 0.011. 3.5-fold increase in water uptake capacity	[45]
4.	Vaccine delivery vehicles(nanoparticles)	Cationic quaternized konjac glucomannan and Anionic carboxymethylated konjac glucomannan	Ovalbumin (OVA)	Boost both humoral and cellular immunological responses Drug loading for TPP/QKGM/OVA and CKGM/QKGM/OVA NPs was 60% and 10.9%, respectively, and the OVA encapsulation efficiency was 49.2% and 67.7%.	[101]
5.	Enzyme encapsulation (hallow nanospheres)	Carboxymethyl konjac glucomannan-grafted poly(ethylene glycol) with α-cyclodextrin complexes	Glucose oxidase (GOX)	Complexed with α-cyclodextrin Greater storage stability, ideal enzymatic activity over a wider pH range, and greater thermostability The encapsulation efficiency was 67.0% at 0.5% concentration of CKGM-g-PEG, with the lowest leakage efficiency of 2%.	[46]
6.	Controlled hydrophobic drug delivery (hydrogel beads)	Carboxymethyl fenugreek galactomannan gellan gum	Glimepiride (GLI)	It improved ex vivo mucoadhesiveness for sustained GLI administration in the therapy of type 2 diabetes. Formulations exhibited extended-release behavior (Q8h, 62–94%) as well as high drug encapsulation efficiency (DEE, 48–97%).	[97]
7.	Sustained drug delivery	Thiolated carboxymethyl chitosan-g-β-cyclodextrin	Ketoprofen	Improved water uptake Five-fold increase in the adhesion time The release behavior of polymer chains is affected by the presence of thiol groups. 35–40% of the drug is released in the first 3 min, followed by a slow and constant flow into the buffer until the establishment of equilibrium.	[70]
8.	Controlled drug delivery(nanoparticles)	Thiolated carboxymethyl chitosan-g-cyclodextrin nanoparticles	Albendazole	Synthesized by anionic-gelation method Thiolation slower release of the entrapped drug, Mucoadhesive drug delivery carrier In the first 10 h, 70% of drugs were released from TGA-CMC-CD, and 78% of drugs were released from TGA-CMC-CD NPs.	[100]
9.	Controlled drug release	Carboxymethyl pullulan, cross-linked with siloxane	Lysozyme, propranolol, quinidine	Enzymes can be immobilized and purified using this method. Cellulose acetate phthalate (CAP)-coated microparticles result in a time-delayed release at both acidic and neutral pH conditions.	[102]
10.	Controlled drug delivery(hydrogel)	Carboxymethyl pullulan, cross-linked with Jeffamine	Lysozyme, BSA, lutein	Polyelectrolyte and thermoassociated properties Hydrogels prepared using Jeff ED-600 showed more hydrophilicity than Jeff ED-2003.	[103]
11.	Colon delivery of drug (matrix tablet)	Calcium-cross-linked carboxymethyl xanthan gum	Prednisolone	The cross-linking resilience had a direct impact on matrix swelling and erosion. The drug release was 25.08%, 50.93%, and 73.70% in 2, 5, and 10 h, respectively. Ca-CMXG matrices with 33.33% (*w*/*w*) and 16.67% (*w*/*w*) CaCl_2_ had lower drug release compared to a CMXG matrix. The matrix with the highest CaCl_2_ concentration i.e., 50% *w*/*w*, was degraded by approximately 55% after 1 h and 74% after 5 h in acid solution.	[84]
12.	Colon delivery of drug (tablet)	Cross-linked chitosan and carboxymethyl guar gum	Fluticasone	Prevents drug absorption in the small intestine and stomach. The swelling was more apparent in IPC films with a 30:70 or 20:80 CH:CMG ratio.	[98]
13.	Colon delivery of drug (Matrix tablet)	Calcium-cross-linked carboxymethyl guar gum	Metronidazole	The viscosity of the gel layer increased with increasing concentrations of Ca^2+^ ions. The release exponent ranged from 0.538 to 0.836 from F1 to F7. The kinetic constant (kr), which reduced from F1 to F4 as the degree of cross-linking increased, ascended with the quantity of calcium.	[104]
14.	Sustained drug delivery (tablet)	Polyacrylamide-grafted carboxymethylated xanthan gum (CMXG-g-PAAm)	--	Release of drugs over a prolonged period of time. The graft copolymerization′s optimal reaction conditions were CMXG = 16 gdm^−3^ (DS = 0.87), AAm = 1 mol dm^−3^; APS = 20 × 10^−4^ mol dm^−3^; reaction time = 75 min and reaction temperature = 75 °C.	[105]
15.	Colon delivery of drug (tablet)	Interpolymer complex films of carboxymethyl fenugreek gum/ carboxymethyl guar gum with chitosan	Tamoxifen	It protected the release of drugs in the small intestine and stomach. CMF:CH (40:60) and CMG:CH (50:50) coating protects drug release in the stomach and small intestine. In the presence of rat cecal components, tamoxifen release was 91% and 94% at pH 6.8.	[39]
16.	Intestine-targeted delivery system	Barium ions cross-linked with carboxymethyl guar gum bipolymeric	Vitamin B12	pH-dependent swelling was found to be 56 ± 4% in SGF (pH 1.2) and 180 ± 8% in SIF (pH 7.4) over the course of 6 h. Sustained for almost 10 h	[106]
17.	Sustained-release drug delivery (multiwalled carbon nanotubes (MWCNTs))	2-hydroxyethyl methacrylate-grafted carboxymethyl guar gum	Diclofenac sodium	Significantly prolong the drug molecules’ half-life (at 1 wt%, 47 h).	[107]
18.	Ocular drug delivery	Cross-linked carboxymethyl sago pulp	Ciprofloxacin	Cross-linked CMSP polymeric chain produced via irradiation resulted in controlled swelling. The disc prolonged the release of the drug in phosphate buffer (pH 7.4) in a first-order fashion for 36 h.	[86]
19.	Sustained-release drug delivery	Al/Ca cross-linked CMTG matrices	Tramadol hydrochloride	The rate of swelling, erosion, and in vitro drug release from Al-CMTG matrices was slower than from Ca-CMTG matrices. The sustained release occurred up to 12 h in the gastrointestinal milieu.	[3]
20.	Oral drug delivery	Poly(ethylene glycol) diacrylate cross-linked carboxymethyl tamarind kernel gum poly(sodium acrylate)	Ciprofloxacin	Enhanced the antimicrobial action of ciprofloxacin	[108]

## Data Availability

Not applicable.
